# Downregulation of MAL2 inhibits breast cancer progression through regulating β-catenin/c-Myc axis

**DOI:** 10.1186/s12935-023-02993-9

**Published:** 2023-07-21

**Authors:** Lijun An, Huiyuan Gong, Xiaojing Yu, Wangming Zhang, Xiaohua Liu, Xiaomin Yang, Liping Shu, Jielin Liu, Liuqi Yang

**Affiliations:** Department of Immunology, Basic Medical College, Guizhou Medical University, Dongqing Road, Guian New District, Guiyang, Guizhou, 550004 China

**Keywords:** MAL2, Breast cancer, Metastasis, Apoptosis, β-catenin/c-Myc axis

## Abstract

**Purpose:**

Myelin and lymphocyte protein 2 (MAL2) is mainly involved in endocytosis under physiological conditions and mediates the transport of materials across the membranes of cell and organelle. It has been reported that MAL2 is significantly upregulated in diverse cancers. This study aimed to investigate the role of MAL2 in breast cancer (BC).

**Methods:**

Bioinformatics analysis and Immunohistochemical assay were applied to detect the correlation between MAL2 expression in breast cancer tissues and the prognosis of breast cancer patients. Functional experiments were carried out to investigate the role of MAL2 in vitro and in vivo. The molecular mechanisms involved in MAL2-induced β-catenin and c-Myc expression and β-catenin/c-Myc-mediated enhancement of BC progression were confirmed by western blot, β-catenin inhibitor and agonist, Co-IP and immunofluorescence colocalization assays.

**Results:**

Results from the cancer genome atlas (TCGA) and clinical samples confirmed a significant upregulation of MAL2 in BC tissues than in adjacent non-tumor tissues. High expression of MAL2 was associated with worse prognosis. Functional experiments demonstrated that MAL2 knockdown reduced the migration and invasion associating with EMT, increased the apoptosis of BC cells in vitro and reduced the metastatic capacity in vivo. Mechanistically, MAL2 interacts with β-catenin in BC cells. MAL2 silencing reduced the expression of β-catenin and c-Myc, while the β-catenin agonist SKL2001 partially rescued the downregulation of c-Myc and inhibition of migration and invasion caused by MAL2 knockdown in BC cells.

**Conclusion:**

These observations provided evidence that MAL2 acted as a potential tumor promoter by regulating EMT and β-catenin/c-Myc axis, suggesting potential implications for anti-metastatic therapy for BC.

**Supplementary Information:**

The online version contains supplementary material available at 10.1186/s12935-023-02993-9.

## Introduction

Breast cancer (BC) is the most common malignancy in women and accounts for 31% of female cancers [1]. Although molecular targeted therapy and immunotherapy have allowed revolutionary changes to BC treatment [2],but the overall progress is slow and clinical translation of this knowledge faced large challenges [3]. In addition, more than 20% of BC patients still develop metastatic disease with a poor prognosis [4]. Therefore, the identification of novel molecular regulators and mechanisms of BC progression will help to find novel targets for BC treatment.

T-cell differentiation protein 2 (Also known as Myelin and lymphocyte protein 2) is a four-pass transmembrane protein composed of 176 amino acid residues. Recent studies have reported that MAL2 acts as an influential regulator in cancers [5], mainly participating in endocytosis under physiological conditions and mediating the transport of intercellular substances [6]. Previous studies have demonstrated increased expression of MAL2 in ovarian cancer [5], prostate adenocarcinoma [7], papillary thyroid cancer [8] and pancreatic cancer [9]. MAL2 has been shown to be associated with the prognosis of patients with pancreatic cancer and colorectal cancer, which can affect the overall survival of patients [10]. Interestingly, a significant association was found between MAL2 expression and the negatively infiltrating level of eosinophils and plasmacytoid dendritic cells [11], and the depletion of MAL2 in breast tumor cells significantly enhanced tumor-infiltrating CD8^+^T cell cytotoxicity and suppressed breast tumor growth, suggesting that MAL2 is a potential immunotherapy target for the treatment of BC [12]. However, the role of MAL2 in BC progression and metastasis remains poorly understood.

Epithelial-mesenchymal transition (EMT) is a cell trans-differentiation process in which epithelial cells acquire mesenchymal characteristics [13]. By activating this EMT program, cancer cells can invade adjacent tissues and migrate to distant organs. EMT progression is regulated by specific components of the major EMT regulators, such as E-cadherin, N-cadherin, vimentin, Snail, SOX2 and OCT4 [14, 15]. Many studies have found that EMT is associated with tumorigenesis, invasion, metastasis and resistance to treatment, especially in BC [16, 17].

The Wnt/β-catenin signaling pathway is involved in many cellular activities, regulating of apoptosis, differentiation, senescence, invasion, migration, and EMT [18, 19]. Upon stimulation with extracellular membrane Wnt, the APC/CK1/GSK-3β/Axin/β-catenin degradation complex was inactivated and the phosphorylation of β-catenin by GSK-3β was inhibited. β-catenin translocations into the nucleus and then interacts with TCF/LEF and activates downstream genes [20], c-Myc is a recognized target gene of β-catenin/TCF transcription factor complex, and is the main carcinogenic driver of tumor growth and metastasis [20]. Moreover, c-Myc contributes to angiogenesis, invasion, and migration [19].

In this study, we report that MAL2 is significantly upregulated in BC tissues compared with the paired noncancerous tissues and that MAL2 overexpression predicts poor prognosis in BC patients. In addition, we observed that knockdown of MAL2 decreased migration and invasion ability as well as increased apoptosis in BC cells. Furthermore, MAL2 downregulation reversed EMT, reduced downstream β-catenin and c-Myc expression in vitro, and inhibited tumor metastatic capacity in vivo. Taken together, our study reveals that MAL2 functions as a novel regulator of BC progression.

## Materials and methods

### Bioinformatic analysis

The transcriptional levels of MAL2 in breast invasive carcinoma (BRCA) and normal breast tissue were obtained from TCGA pan-cancer view by using the GEPIA database (http://gepia.cancer-pku.cn) and UALCAN database (http://ualcan.path.uab.edu/). Kaplan-Meier survival analysis was used to evaluate the prognostic value of MAL2 genes. According to the median of the expression of MAL2, we divided patients into highly expressed group and lowly expressed group.

### Clinical samples and immunohistochemistry staining

Tissues samples containing 20  BC tissues, 15 fibroadenoma tissues and 13 paracancerous tissues were collected from The First Hospital of Guizhou medical University with informed consent. None of the recruited BC patients received chemotherapy, radiotherapy or biological therapy. All of the patients and their families signed the informed consent. The whole process obeyed the rules of the Ethics Committee of the Guizhou medical University.

Tissue sections were baked at 60°C for 1 h, dewaxed in xylene, rehydrated through a gradient concentration, and the endogenous peroxidase activity was blocked with 3% hydrogen peroxide. After antigen retrieving by citrate buffer using a microwave oven, the sections were incubated with the primary antibody MAL2 (purchased from BIOSS, BS-7175r, 1: 200 dilution) at 4 °C overnight. Then, tissue sections were incubated with primary antibody-derived secondary antibody (purchased from proteintech, SA00001-2, 1:2000). Finally, the sections were visualized after staining with DAB and counterstained with haematoxylin. IHC staining score was assessed by pathologists who were blinded to the patients’ clinicopathological information. The scoring criteria according to the intensity of staining are as follows: negative (unstained), 0 points; weakly positive (yellow), 1 point; moderately positive (brown), 2 points; and strongly positive (brown), 3 points. Percentage of positively stained tumor cells was scored as follows: 1 (< 10%), 2 (10–50%), 3 (50–75%), and 4 (> 75%). The protein IHC staining index for each section was the product of the staining intensity score and the positive cell proportion score.

### Cell culture

BC cell lines (MDA-MB-231, MDA-MB-453, MDA-MB-468) and human normal breast epithelial cells (MCF-10 A) were obtained from Shanghai Cell Bank, Chinese Academy of Sciences. MCF-7 cells were stored by the laboratory of Immunology, Guizhou Medical University. MDA-MB-231 and MDA-MB-453 cells were cultured in L15 medium supplemented with 10% fetal bovine serum and 1% penicillin and streptomycin; MCF-7 and MDA-MB-468 cells were cultured in DMEM medium supplemented with 10% fetal bovine serum and 1% penicillin and streptomycin; MCF-10 A cells were cultured in special complete medium.

### Transfection

The short interfering RNAs (siRNA) were synthesized by GenePharma (Shanghai, China). The MAL 2 and the negative control siRNAs were transfected into the cells using a Lipofectamine 3000 (Invitrogen, USA) according to the manufacturer’s suggestions. Sequences for siMAL2 (GenePharma) were as follows: si MAL2 #1: 5’-GGUGGCUCAAAUUGAUGCUTT-3’; si MAL2 #2: 5’-CCUGAGUGAUAACCAGUAUTT-3’; si MAL2 #3: 5’-GGUCUGGCUUUACGAAGAUTT-3’; siMAL2-NC: 5’-UUCUCCGAACGUGUCACGUTT-3’. To establish stably transfected cells, the cells were transfected with an MAL2 shRNA using polybrene according to the manufacturer’s instructions and then selected in puromycin (2 µg/mL for MDA-MB-231 and 2.0 µg/mL for MCF-7, GeneChem, Shanghai, China) for 48 h. The infection efficiency was observed under inverted fluorescence microscope, and the silencing effect was detected by western blotting.

### Wound healing assay

After incubation for 24 h, the cells grow to 90-100% for scratching. The cell monolayers were scratched with a 200 µL pipette tip. After washing with PBS, the cells were cultured with serum-free medium and allowed to migrate for 24 h. Images were acquired at 0 h,12 and 24 h and then analyzed with Image J software.

### Transwell migration and invasion assay

24-well transwell chambers with 6.5-µm pore size polycarbonate (corning) were used to test cell invasive and migratory ability. Briefly, 7.5 × 10^4^ infected cells in serum-free DMEM/L15 medium were transferred into the upper chamber of an insert with Matrigel or not, and DMEM/L15 medium supplemented with 10% FBS was added to the lower chamber. After incubation for 24 h, the cells remaining on the upper membrane were removed with cotton wool, and the cells that had migrated or invaded through the other side of the membrane surface were fixed with methanol and stained with 0.1% crystal violet (Solarbio). Three-Five random fields were imaged and counted under an inverted microscope.

### Flow cytometry

Cell culture medium and cells were collected and washed with pre-cooled PBS, and the supernatant was discarded. The cells were suspended with 500 µL 1×binding buffer solution. 10 µL 7-AAD and 5 µL Annexin V-APC were added to each cell sample. After mixing and staining for 10 min at room temperature, cells were analyzed with a FACScan® flow cytometer (BD Biosciences) equipped with CellQuest software (BD Biosciences). Cells were classified into viable cells, dead cells, early apoptotic cells and late apoptotic cells, and the relative ratio of early and late apoptotic cells was compared with control from each experiment.

### Acridine orange/ethidium bromide staining

The cells were resuspended with PBS after collection, and AO/EB solution was prepared afterwards (reagent A: reagent B: reagent C = 1:1:8). We added 1 µL of AO/EB working solution every 15 µL cell suspension, mixed them, incubated them for 15 min at room temperature, and viewed them under a fluorescence microscope. Dead cells fluoresce orange, while living cells fluoresce green.

### Western blot

Cells were harvested and lysed with RIPA and the protein concentration was detected by BCA protein assay (Solarbio,PC0020). Twenty mg total proteins were separated by 10% SDS-PAGE and transferred to PVDF membrane, then incubated with the primary antibodies against E-cadherin (BA0475, 1:1500, BOSTER, China), N-cadherin (BA0673, 1:1000, BOSTER, China), Vimentin (PB9359, 1:1500, BOSTER, China), Bax (380,709, 1:2000, ZenBio, China), Bcl2 (381702,1:2000ZenBio, China), Cleaved-Caspase-3p17 (341,034, 1:1000ZenBio, China), Cleaved-Caspase-8-p18 (251,941, 1:2000ZenBio, China), β-catenin (R22820, 1:2000 ZenBio, China), c-Myc (R22809, 1:2000 ZenBio, China), Caspase-3 (R23315, 1:2000 ZenBio, China), MAL2 (BS-7175r, 1:1500, BIOSS BIOSS, China), GAPDH (GB11002, 1:2000, Servicebio, China) overnight. The membranes were subsequently incubated with the appropriate HRP-conjugated secondary antibodies (purchased from proteintech, SA00001-2, 1:10000) for 1.5 h, and signals were visualized using an ECL detection system.

### Co-immunoprecipitation

According to the DIA IP/CoIP Kit (KM0134) manufacturer’s instructions, cells were collected and lysed for 20 min. Cell lysates were centrifuged at 12,000 rpm for 10 min at 4℃. Then, the beads were washed 3 times with PBS buffer, the MAL2 antibody (BS-7175r,1:50), β-catenin (ZEN, 1:50) or IgG and beads were turned over for 20 min at room temperature, after which the bead-antibody mixture was washed with PBST five times. The supernatant containing proteins was resuspended with the bead-antibody mixture, incubation was then reversed at 4℃ for more than 8 h. Then, the bead-antibody-antigen mixture was washed with PBST five times, resuspended with 1x loading buffer, and heated at 100℃ for 5 min. The supernatant was used for western blotting.

### Immunofluorescence analysis

Cells were fixation with 4% paraformaldehyde for 20 min. The fixed cells were permeabilized 10 min with 0.2% Tween-20,washed with PBS and blocked with PBS containing 5% BSA for 30 min. Immunostaining was done by incubating the samples successively with antibodies specifically recognizing β-catenin, MAL2 and fluorescein-conjugated secondary antibody. The nuclei were then restained with DAPI. The fluorescence was examined under a fluorescencemicroscope (Nikon ci-e-ds-r11).

### Nude mouse lung metastasis assay

Twelve nude mice (BALB/C, 4-week-old, female) were purchased from Beijing Huafukang Biosciences (Experimental Animal Production License No: SCXK(Beijing) 2019-0008). The animal experiments met the requirements of the Animal Care and Use Committee of China Medical University. The mice were divided into two groups of 6 mice each, and 8 × 10^5^ MDA-MB-231 cells with stable knockdown of MAL2 or control cells were injected via the tail vein. After six weeks, mice were sacrificed by cervical dislocation under anesthesia, the lung tissue was harvested, the number of nodules was counted, and the lesions were observed by HE staining. The animal experiment has been approved by the Animal Experiment Ethics Committee of Guizhou Medical University.

### Statistical analysis

Data are presented as the mean ± standard deviations (SD). SPSS22.0 software was used to perform the statistical analysis. The Student’s t-test was performed to assess the values between two groups, and Analysis of Variance (ANOVA) was performed for analysis among multiple groups. The survival rate of BC patients was analyzed by applying Kaplan–Meier method and calculated with log-rank test. *P* < 0.05 was considered statistically significant. (*P < 0.05, **P < 0.01, and ***P < 0.001).

## Results

### MAL2 is highly expressed in BC tissue and cells

**Fig. 1:**
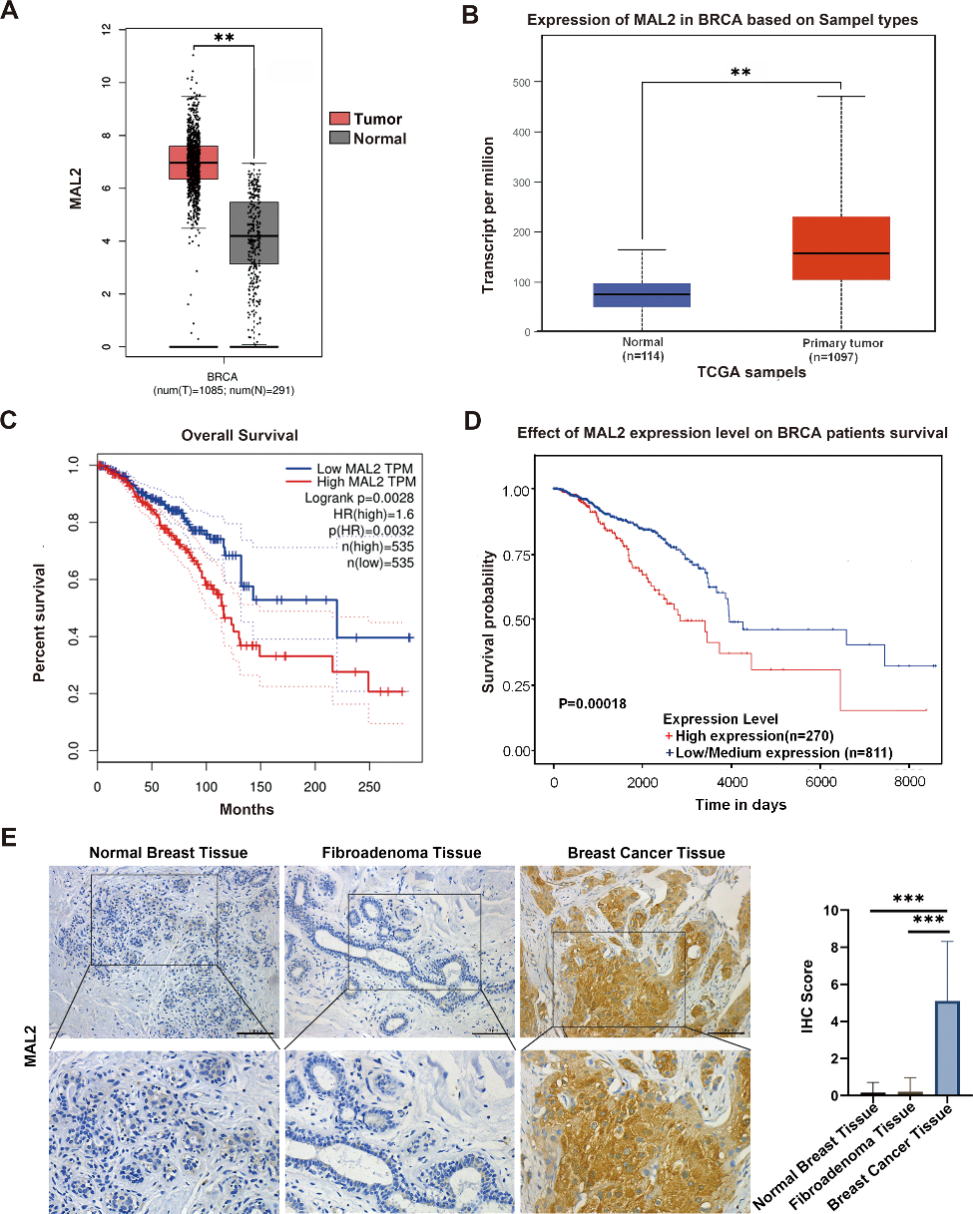
**MAL2 is upregulated in BC tissues and is associated with poor prognosis. A** Bioinformatic analyses of the MAL2 expression in BC tissues (n = 1085) and adjacent noncancerous tissues (n = 291) from GEPIA datasets. **B** The expression level of MAL2 in primary tumor tissues (n = 1097) and adjacent noncancerous tissues (n = 114) from the UALCAN database. **C** Kaplan-Meier analysis of the correlation between the MAL2 expression level and the overall survival of BC patients represented in the tissue array from GEPIA datasets. **D** Kaplan-Meier analysis of the correlation between the MAL2 expression level and the overall survival of BC patients represented in the tissue array from UALCAN database. **E** Representative immunohistochemical (IHC) staining images of MAL2 expression in BC (n = 21), fibroadenoma (n = 16) and adjacent normal tissues (n = 13). The scale bars in the 200× magnification images represent 100 μm. All data are represented as mean ± SD. ***P < 0.001

To assess the role of MAL2 in BC, we first analyzed the MAL2 expression level in 1085 BC tissues and 291 normal tissues using GEPIA database and 1097 BC tissues and 114 normal tissues using UALCAN database. We found that MAL2 was significantly upregulated in BC tissues compared to the nontumor tissues (Fig. 1A and B; *P < 0.01*). Kaplan-Meier analysis revealed that patients with a high MAL2 expression exhibited shorter survival compared with patients with a low MAL2 level (Fig. 1C and D; *P < 0.01*). To further validate the protein expression of MAL2 in BC, immunohistochemistry was used to detect MAL2 in samples of 20 BC tissues, 13 samples of normal breast tissues and 15 samples of fibroadenoma tissues. It was found that MAL2 expression in BC tissues was significantly increased in comparison with that in noncancerous tissues (Fig. 1E; *P < 0.01*). These results demonstrate that MAL2 is significantly upregulated in BC tissues suggesting MAL2 may function as a tumor-promoting factor in human BC.

**Fig. 2:**
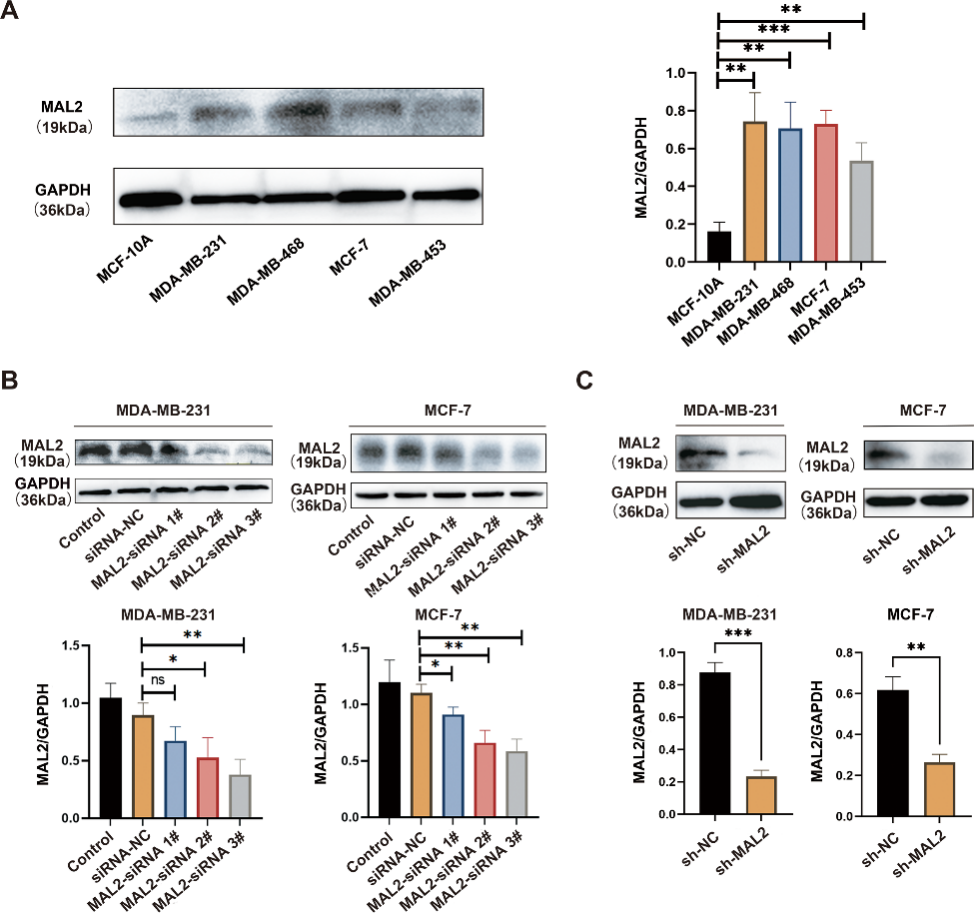
**MAL2 is upregulated in BC cells. A** Western blot analysis of MAL2 expression in BC cell lines and a normal human mammary epithelial cell line (MCF-10 A). **B** Western blot assay quantifies the knockdown efficiency of MAL2 expression in MDA-MB-231 and MCF-7 cells. **C** The protein levels of MAL2 in sh-NC and sh-MAL2-treated MDA-MB-231 and MCF-7 cells. All data are represented as mean ± SD. *P* < 0.05; *P < 0.01;***P < 0.001; ns, not significant

Western blot showed that the expression of MAL2 was significantly higher in a panel of BC cell lines than that in the noncancerous breast cell line (MCF-10 A) (Fig. 2A). Based on the high expression of MAL2, human MDA-MB-231 and MCF-7 cells were selected for further MAL2 studies in BC. To find out the possible role of MAL2 in BC in vitro, MAL2 was knocked down in BC cell line MDA-MB-231 and MCF-7 cells by transfection with specific MAL2 siRNA. In addition, western blot was performed to detect MAL2 expression in MDA-MB-231 and MCF-7 cells transfected with different MAL2 siRNAs for 48 h. Greater knockdown efficiency was observed for using MAL2-siRNA 3# as compared to using MAL2-siRNA 1# and MAL2-siRNA 2# (Fig. 2B), MAL2-siRNA 3# was therefore selected to construct the shRNA interference vector lentivirus and stable low-expression cell lines. The western blot results showed that MAL2 knockdown by shRNA significantly reduced MAL2 expression as compared to the control (Fig. 2C).

### MAL2 downregulation inhibits migration, invasion and EMT

**Fig. 3:**
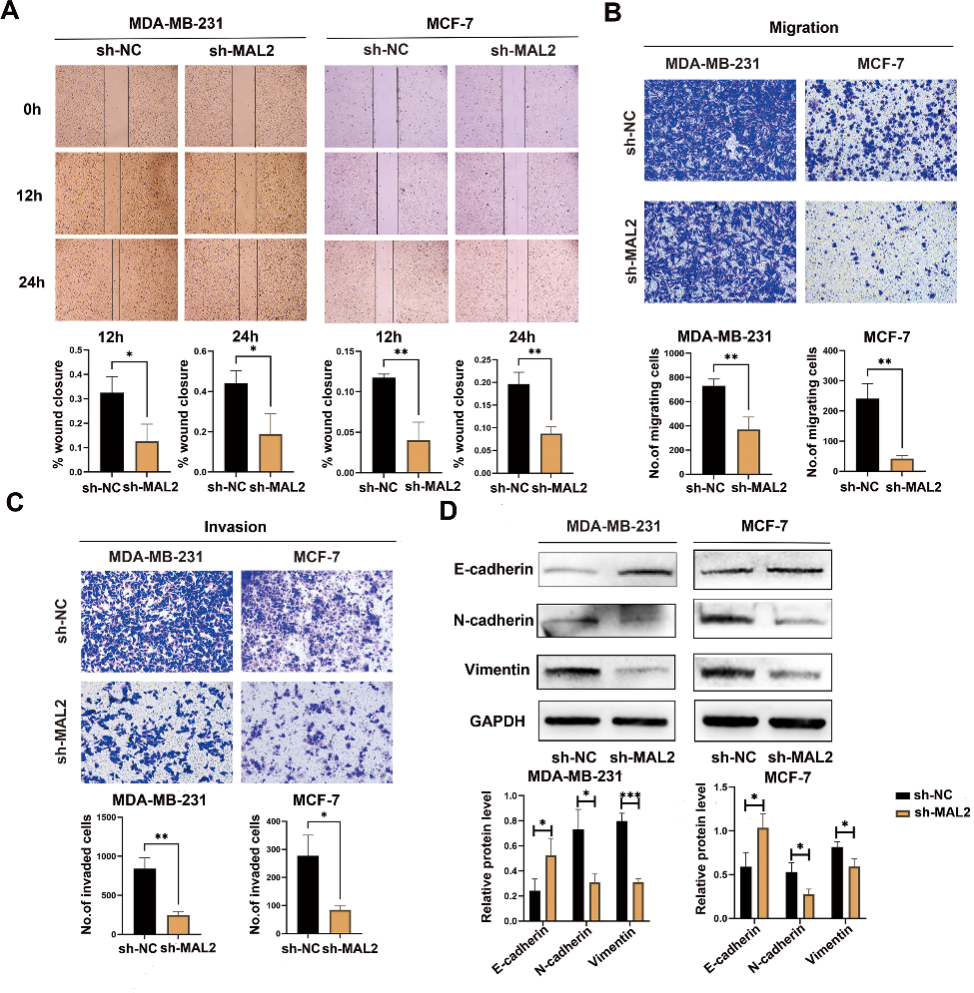
**MAL2 downregulation inhibits migration, invasion and EMT. A** The migration of MDA-MB-231 and MCF-7 cells transfected with sh-NC and sh-MAL2 was evaluated by wound healing assays. **B** The cell migration of MDA-MB-231 and MCF-7 cells infected with sh-NC and sh-MAL2 was examined by transwell assays. **C** The invasion of MDA-MB-231 and MCF-7 cells transfected with sh-NC and sh-MAL2 was analyzed by transwell assays. **D** Western blot analysis was performed to measure the expression of EMT marker in BC cells. All data are represented as mean ± SD. * *P* < 0.05; ** *P* < 0.01; *** *P* < 0.001; ns,not significant

To unravel the biological function of MAL2 in BC cells, the viability of cell migration and invasion were examined using the wound healing and transwell assays after knockdown of MAL2. The results of wound healing and transwell migration assays revealed that knockdown of MAL2 markedly reduced migration ability compared with the control group (Fig. 3A and B). Transwell invasion assay was used to assess the invasion abilities of MDA-MB-231 and MCF-7 cells. Our results unveiled that silencing MAL2 reduced the number of cells that invaded the membrane of BC cells (Fig. 3C). In addition, the effects of MAL2 knockdown on EMT were examined by detecting the expression of EMT markers E-cadherin, N-cadherin and Vimentin. Western blot results showed that the expression of N-cadherin and Vimentin decreased, while the expression of E-cadherin increased in MDA-MB-231 and MCF-7 cells following MAL2 knockdown (Fig. 3D). Taken together, these results suggest that knockdown of MAL2 inhibits migration, invasion and EMT of BC cells.

### MAL2 downregulation induces apoptosis of BC cells

**Fig. 4:**
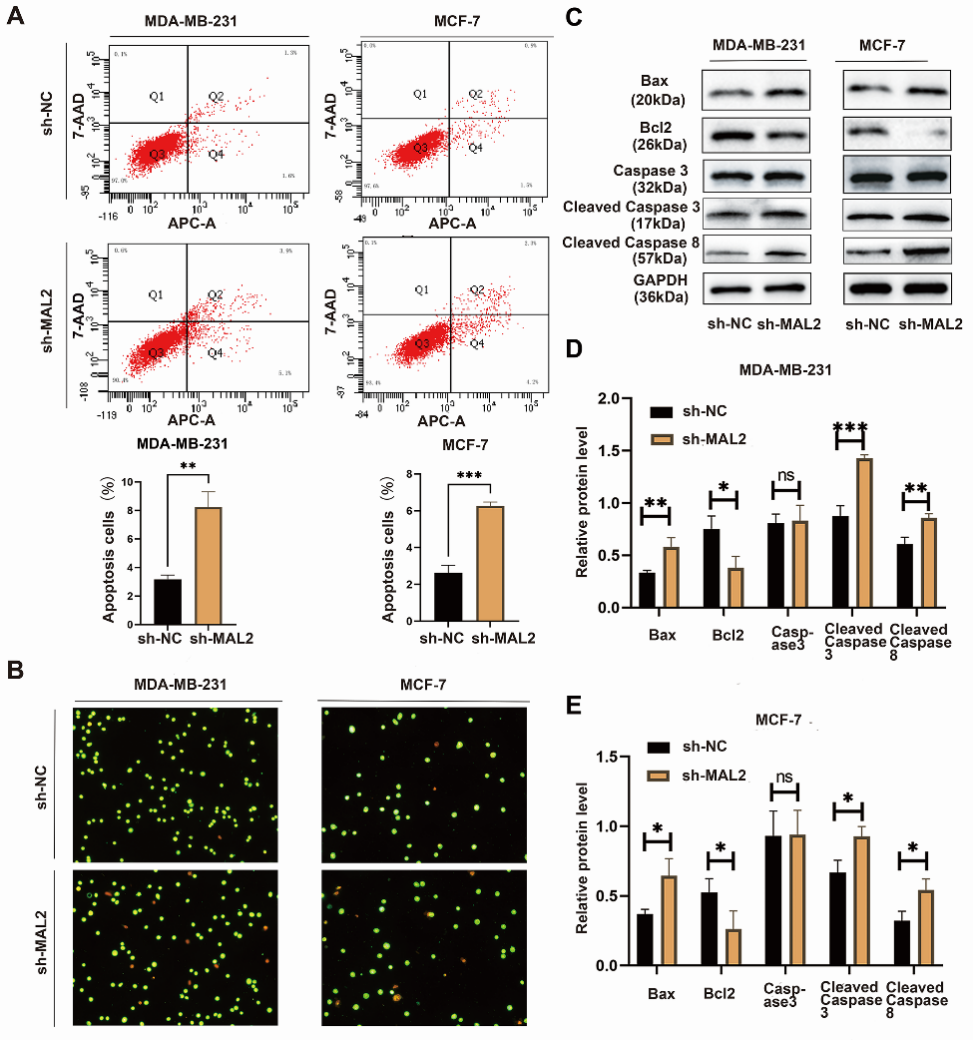
**MAL2 downregulation induces apoptosis of BC cells. A** Flow cytometric analysis detects apoptosis of MDA-MB-231 and MCF-7 cells transfected with sh-NC and sh-MAL2. **B** AO-EB double staining evaluates apoptosis of MDA-MB-231 and MCF-7 cells transfected with sh-NC and sh-MAL2. **C** Western blot assay was performed to measure the expression of apoptosis-related proteins in MDA-MB-231 and MCF-7 cells after MAL2 knockdown. **D** The relative expression level of apoptosis-related proteins in MDA-MB-231 cells. **E** The relative expression level of apoptosis-related proteins in MCF-7 cells. All data are expressed as mean ± SD. * *P* < 0.05; ** *P* < 0.01;*** *P* < 0.001; ns, not significant

To further investigate the effect of MAL2 gene knockdown on apoptosis of MDA-MB-231 and MCF-7 cells, flow cytometry analysis and AO-EB double staining were performed. Flow cytometry results demonstrated that the total apoptotic rate of MDA-MB-231 and MCF-7 cells with sh-MAL2 was higher than that of cells in sh-NC group (Fig. 4A). AO-EB double staining also proved that the number of apoptotic cells in MAL2 silencing group was higher than that in sh-NC group (Fig. 4B). At the molecular level, we detected apoptosis-related proteins, and the results showed that after MAL2 knockdown, the protein expressions of Bax, Cleaved-caspase 3, and Cleaved-caspase 8 were significantly increased, while the expression of Bcl2 decreased (Fig. 4C, D and E). These results indicated that MAL2 knockdown could induce apoptosis of BC cells.

### MAL2 downregulation inhibits BC lung metastasis

**Fig. 5:**
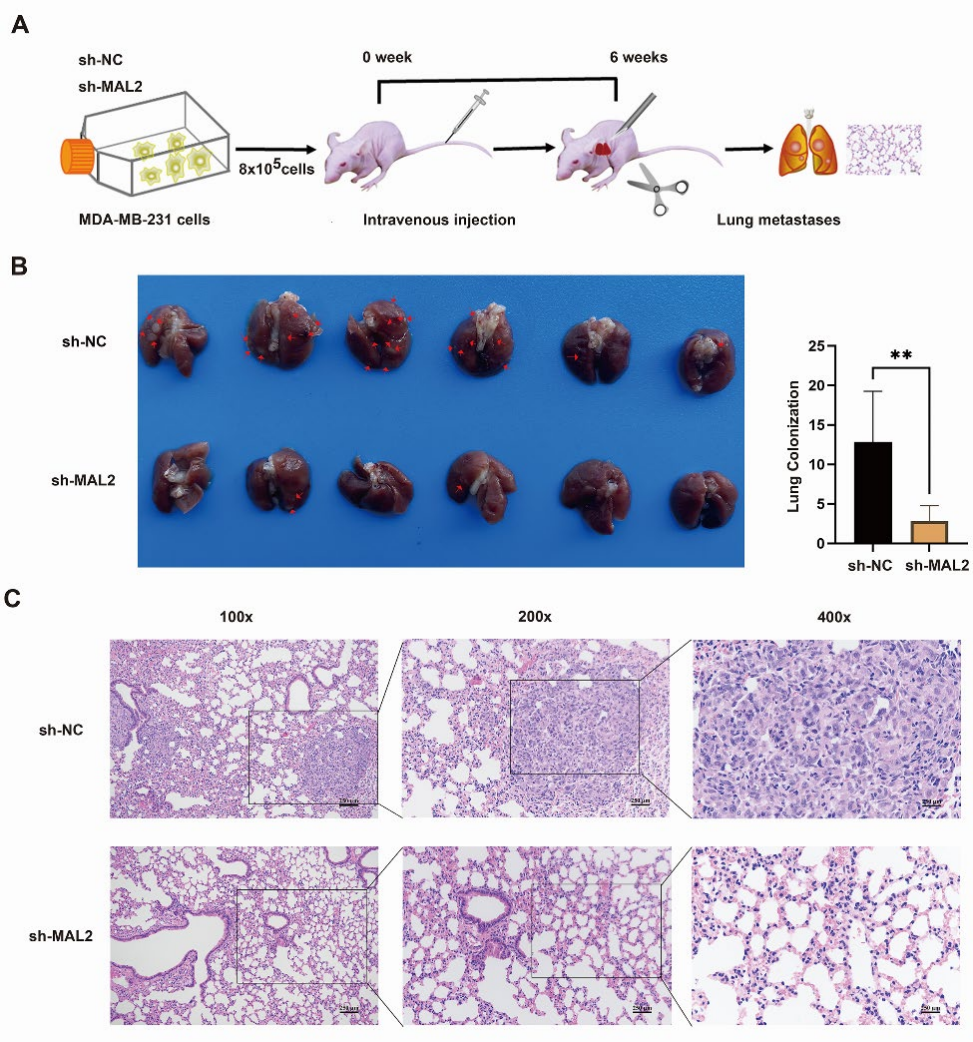
**MAL2 downregulation inhibits BC lung metastasis in vivo. A** Schematic flowchart of the MDA-MB-231 cells in vivo metastasis model. **B** Representative photographs of lung metastatic nodules developed in mice 6 weeks after injection of MAL2-silencing or control MDA-MB-231 cells. Red arrowheads denote metastatic nodules established in lungs. **C** Representative HE images of nude mouse lung 6 weeks following injection of MDA-MB-231 sh-NC and sh-MAL2. Scale bar: 250 μm. All data are expressed as mean ± SD. ** *P* < 0.01

Since MAL2 downregulation was found to inhibit the migration and invasion of BC cells in vitro, we next explored the possible role of MAL2 in lung metastasis of BC cells. BALB/c nude mice were intravenously (i.v.) injected sh-NC or sh-MAL2-transfected MDA-MB-231 cells. Two groups of mice were killed without pain 6 weeks after inoculation, and their lungs were surgically excised and subjected to detection of metastatic lung lesions (Fig. 5A). Our results showed that the lungs of MDA-MB-231 cells transfected with sh-MAL2 produced fewer nodules than the sh-NC group (Fig. 5B). H&E staining of lung tissue sections was found that the normal alveolar tissue in the sh-NC group was more disappeared and diseased, and the diseased part accounted for more, while the normal part of the alveolar tissue in the sh-MAL2 group was more,and the diseased part was less(Fig. 5C). These findings demonstrate that MAL2 downregulation inhibits BC lung metastasis in vivo.

### MAL2 regulates β-catenin/c-Myc

**Fig. 6:**
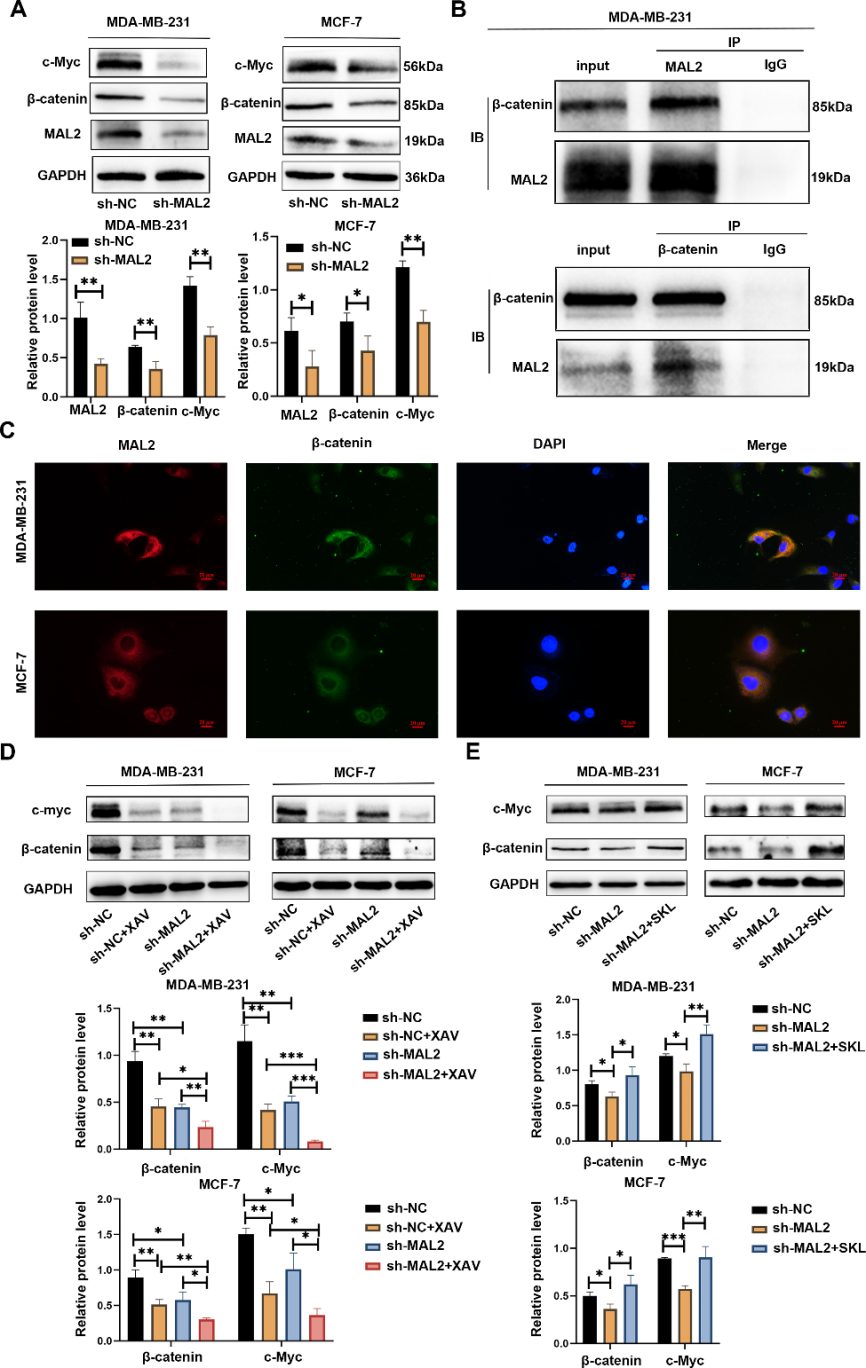
**MAL2 regulates β-catenin /c-Myc signaling. A** Western blot analysis of MAL2, β-catenin and c-Myc expression in BC cells after silencing MAL2. **B** Immunoprecipitation was performed with MDA-MB-231 cell lysates using MAL2 or β-catenin antibodies, and the precipitants were detected by western blot with the indicated antibodies. **C** Double immunostaining of MAL2 (red) and β-catenin (green) in the MDA-MB-231 cells. Scale bar: 20 μm. **D** MDA-MB-231and MCF-7 cells infected with sh-NC and sh-MAL2 were treated with β-catenin inhibitor XAV-939 (20 µmol/L) for 24 h. **E** MDA-MB-231and MCF-7 cells infected with sh-NC and sh-MAL2 were treated with β-catenin agonist SKL2001 (20 µmol/L) for 24 h. The protein expression of β-catenin and c-Myc were evaluated by western blot assay. All data are expressed as mean ± SD. * *P* < 0.05; ** *P* < 0.01; *** *P* < 0.001

Bioinformatics analysis in a previous study suggested that the high expression of MAL2 in BC enriches MYC target V1. The abnormal expression of c-Myc is generally considered to be closely related to cell migration and invasion, and β-catenin is an important factor to activate c-Myc expression in cancer cells. Thus, we aimed to investigate the relevance of MAL2, c-Myc and β-catenin in BC cells. The results showed that c-Myc and β-catenin expression was downregulated when MAL2 was silenced in MDA-MB-231 and MCF-7 cells (Fig. 6A). Consistent with the intracellular localization of MAL2, β-catenin is also expressed predominantly in the cell membrane and cytoplasm and is involved in cell-cell adhesion. Thus, we firstly identified the interaction between MAL2 and β-catenin in BC cells. The results of immunoprecipitation of whole cell lysates from MDA-MB-231 cells showed that MAL2 interacted with β-catenin in vitro (Fig. 6B). We also performed double immunofluorescence staining of MAL2 and β-catenin in MDA-MB-231 cells, and the results showed that the fluorescence signals of MAL2 and β-catenin were mainly colocalized in the cell cytoplasm and membrane (Fig. 6C), further suggesting an interaction between MAL2 and β-catenin. To explore whether β-catenin and c-Myc are correlated, we treated MAL2-knockdown cells with pharmacological β-catenin inhibitor, XAV-939, and subsequently measured β-catenin and c-Myc expression. The results showed that the expression of c-Myc was downregulated along with the downregulation of β-catenin (Fig. 6D). To explore whether MAL2 regulates the expression of c-Myc by β-catenin, MAL2-knockdown cells were treated with β-catenin agonist SKL2001 (SKL), and β-catenin and c-Myc expression were subsequently measured. As shown in Fig. 6E, β-catenin agonist rescued MAL2-induced downregulation of β-catenin and c-Myc. These results suggested that c-Myc expression regulated by MAL2 is dependent on β-catenin.

### Stabilizing β-catenin rescues the inhibitory effect of MAL2 downregulation on migration, invasion and EMT

**Fig. 7:**
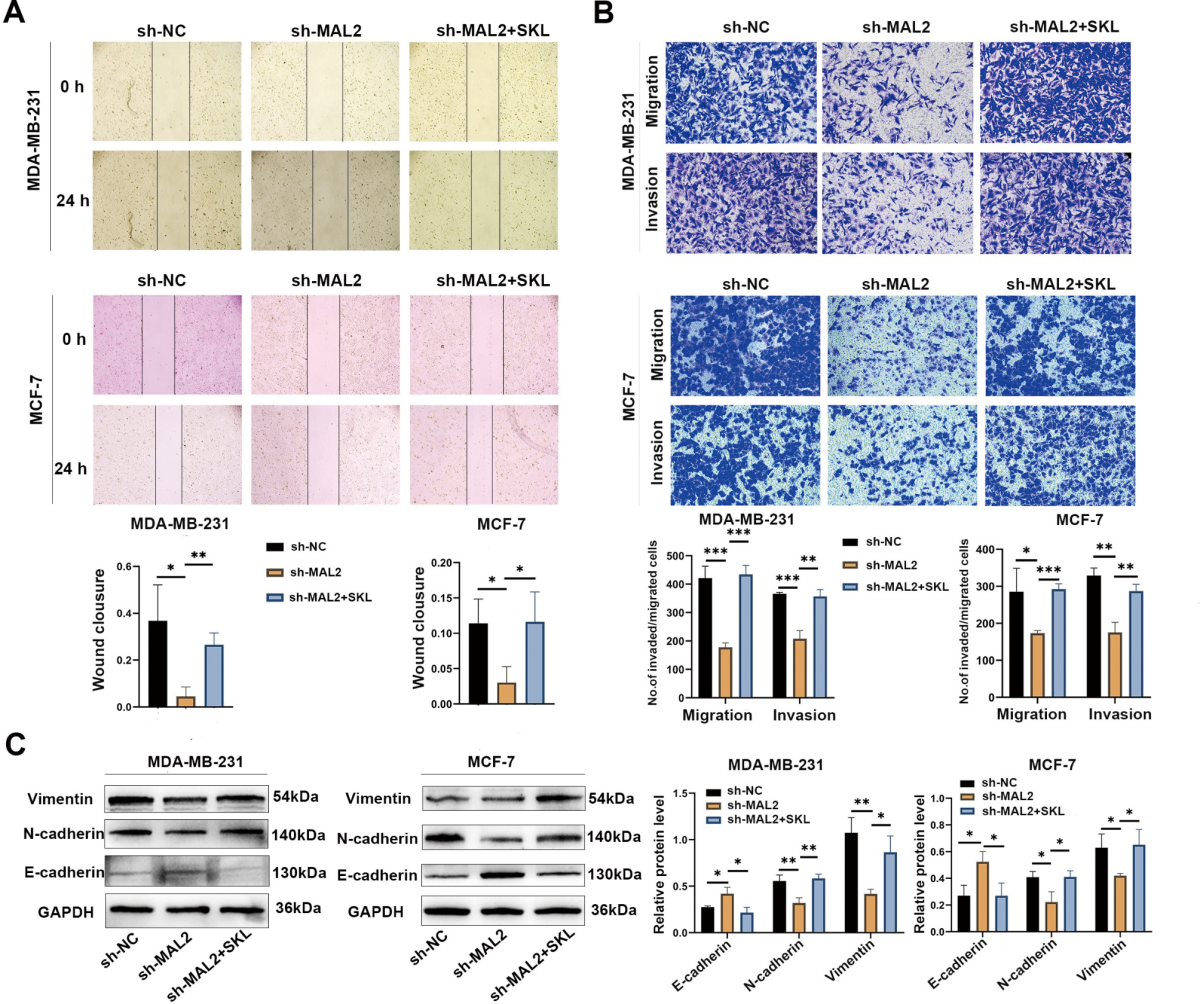
**Stabilizing the β-catenin rescues the inhibition of migration and invasion caused by MAL2 knockdown. A** The wound healing ability of MDA-MB-231 and MCF-7 cells infected with sh-NC and sh-MAL2 was determined by scratch assay after treatment with SKL2001(20 µmol/L). **B** Transwell migration and invasion assays were used to detect the migration and invasion ability of MDA-MB-231 and MCF-7 cells infected with sh-NC and sh-MAL2 after treatment with SKL2001 for 24 h (20 µmol/L). C Wetern blot was used to detect the expression of EMT-related proteins in MDA-MB-231 and MCF-7 cells infected with sh-NC and sh-MAL2 after 24 h SKL2001 (20 µmol/L) treatment. All data were presented as mean ± standard deviation. *P < 0.05; **P < 0.01; ***P < 0.001; n = 3

SKL2001 protects β-catenin from proteasomal degradation by inhibition of phosphorylation at residues Ser 33/37/ Thr 41 and Ser 45. We therefore performed rescue experiments using the β-catenin agonist SKL2001 to investigate whether MAL2 regulates the invasion and metastasis of breast cancer cells via the β-catenin/c-Myc axis. The results of wound healing assay showed that SKL2001 treatment partially saved the diminished wound healing ability caused MAL2 silencing (Fig. 7A). In addition, BC cells treated with SKL2001 for 24 h were harvested for transwell migration and invasion assay. As shown in Fig. 7B, BC cells exhibited increased migration and invasion ability in the MAL2 silencing + SKL2001 group as compared to the MAL2 silencing group alone (*P* < 0.05)., Furthermore, the EMT-associated proteins were detected in MDA-MB-231 and MCF-7 cells after SKL2001 treatment for 24 h. The results showed that the down-regulation of N-cadherin and Vimentin induced by MAL2 silencing were partially reversed (Fig. 7C), indicating that stabilizing the β-catenin could partially rescue the inhibitory effect of MAL2 downregulation on migration, invasion and EMT progression of BC cells.

## Discussion

MAL2 has been identified as a mediator of various pathological conditions, including cancers. Study have shown that high expression of MAL2 facilitates the proliferation of lung cancer cells in vitro and in vivo [21]. Furthermore, knockout of MAL2 inhibits the proliferation, invasion and migration and promotes apoptosis of ovarian cancer (OC) cells in vivo and in vitro [5]. Nevertheless, recent studies suggest that MAL2 may be a promising target for cancers such as colorectal cancer [22] and hepatocellular carcinoma [23].Therefore, the function of MAL2 in different cancers are inconsistent, indicating that the role of MAL2 may be organ-dependent. Although Bhandari et al. reported that MAL2 was able to promote BC proliferation, migration and invasion [24], the detailed biological functions of MAL2 in BC progression and the underlying mechanisms are not well understood. Our study explored the role of MAL2 in migration and invasion ability of BC cells using MDA-MB-231 and MCF-7 cells in vitro. Although MCF-7 is ER/PR positive cells, it expresses more MAL2 in cells as MDA-MB-231 cells. Moreover, the selection of a triple-negative breast cancer cell line and a non-triple-negative breast cancer cell as the study samples would make our study samples more widely representative. The results showed that MAL2 knockdown could inhibit cell migration and invasion and promote apoptosis of MDA-MB-231 and MCF-7 cells, suggesting that MAL2 regulates the migration and invasion of breast cancer cells probably through a pathway that is not associated with ER/PR/HER2. As an important intracellular carrier of vesicle trafficking, MAL2 mainly involved in endocytosis and mediates intercellular material transport. Thus, inhibition of MAL2 expression in BC cells may inhibit the transmembrane transport of substances required for the BC progression.

Tumor metastasis is usually involved in the EMT [25]. Activation of this EMT program confers cancer cells the potential to inhibit epithelial genes that promote cell adhesion (adhesion junctions, tight junctions, and desmosomes) and invade adjacent tissues [26]. The classical epithelial marker E-cadherin (CDH1) is a key component of adhesion and is the most significant inhibitory target in the process of EMT [27]. Cells undergoing EMT must activate mesenchymal genes, including N-cadherin and vimentin, to promote the morphological and behavioral changes required for migration [17]. In our study, the upregulation of E-cadherin, downregulation of N-cadherin and Vimentin were found after MAL2 knockdown, suggesting that MAL2 may be involved in the EMT process. In addition, through the construction of lung metastasis model of BC, we found that MAL2 knockdown could inhibit the number of nodules transferred from BC to lung, suggesting that MAL2 may play an important role in the BC metastasis.

The enrichment plots of GSEA in BC in previous study showed that the MYC targets V1 was obviously upregulated with a high-MAL2-expression [11]. The c-Myc is a gene highly correlated with cancer that is involved in tumor initiation and progression. Moreover, c-Myc contributes to angiogenesis, invasion, and migration [19, 28]. Recently, many studies have shown that the transactivation of c-Myc is regulated by up-stream cytokine signaling, transcription factors and related binding proteins, among which β-catenin is an important factor in c-Myc activation [29]. Beta-catenin is a dual functional protein that both mediates cell-to-cell adhesion at adhesion junctions and regulates the transcription of target genes. Beta-catenin forms a complex with E-cadherin, which in turn, can function as an anchoring junction and act to stabilize cell adhesion [30, 31]. GO enrichment analysis revealed that MAL2 mainly mediated in cadherin binding which involved in cell-cell adhesion and epidermis development [5]. In addition, β-catenin is mainly expressed in the cell membrane and cytoplasm where MAL2 is located. Thus, we hypothesized that MAL2 might have some interaction with β-catenin. The results of Co-IP and double immunofluorescence assay confirmed the speculation of an interaction between MAL2 and β-catenin. Since the correlation between β-catenin and c-Myc has been widely reported, we used β-catenin inhibitors and agonists to verify the association between β-catenin and c-Myc. Western blot results showed that, a more significant downregulation of c-Myc was observed after a combination of the MAL2 silencing and β-catenin inhibitors XAV-939. In contrast, β-catenin agonist SKL2001 rescued MAL2-induced downregulation of β-catenin and c-Myc expression. SKL2001 can up-regulate β-catenin-regulated transcription by disrupting β-catenin and Axin interaction, thereby preventing β-catenin phosphorylation (Ser33/Ser37/Thr41/Ser45) and proteasomal degradation [32]. These results suggested that MAL2 induced c-Myc expression dependent on β-catenin. In addition, the results of cell scratch, migration and invasion and western blot showed that SKL2001 treatment after MAL2 knockdown could partially reverse the effects of MAL2 silencing on migration, invasion and EMT progression, suggesting that β-catenin was involved in the regulation of MAL2 on BC progression. In summary, our results show that MAL2 regulates apoptosis, invasion and metastasis in BC via the β-catenin/c-Myc axis. However, the study of the interaction between MAL2 and β-catenin is insufficient, and the related mechanism needs to be further explored.

In conclusion, MAL2 plays a potential role in BC metastasis and serves as a tumor promoter in BC cells. MAL2 knockdown inhibited cancer cell migration, invasion, and promoted apoptosis in vitro, and inhibited tumor metastasis in vivo possibly by regulating the EMT and β-catenin/c-Myc pathway. Further progress in understanding the mechanism of MAL2 action in BC may be needed. Taken together, our study indicates that MAL2 could be a unique future therapeutic target for controlling the progression and metastasis of BC.

## Electronic supplementary material

Below is the link to the electronic supplementary material.

Supplementary Material 1

## Data Availability

All data needed to evaluate the conclusions in the paper are presented in the paper and/or the Additional files. Additional data related to this paper may be requested from the authors.
